# Direct dyes removal using modified magnetic ferrite nanoparticle

**DOI:** 10.1186/2052-336X-12-96

**Published:** 2014-06-11

**Authors:** Niyaz Mohammad Mahmoodi, Jafar Abdi, Dariush Bastani

**Affiliations:** 1 Department of Environmental Research, Institute for Color Science and Technology, Tehran, Iran; 2 Department of Chemical Engineering, Sharif University of Technology, Tehran, Iran

**Keywords:** Magnetic nanoparticle, Adsorbent, Dye removal, Modification, Colored wastewater

## Abstract

The magnetic adsorbent nanoparticle was modified using cationic surface active agent. Zinc ferrite nanoparticle and cetyl trimethylammonium bromide were used as an adsorbent and a surface active agent, respectively. Dye removal ability of the surface modified nanoparticle as an adsorbent was investigated. Direct Green 6 (DG6), Direct Red 31 (DR31) and Direct Red 23 (DR23) were used. The characteristics of the adsorbent were studied using Fourier transform infrared (FTIR), scanning electron microscopy (SEM) and X-ray diffraction (XRD). The effect of adsorbent dosage, initial dye concentration and salt was evaluated. In ternary system, dye removal of the adsorbent at 90, 120, 150 and 200 mg/L dye concentration was 63, 45, 30 and 23% for DR23, 97, 90, 78 and 45% for DR31 and 51, 48, 42 and 37% for DG6, respectively. It was found that dye adsorption onto the adsorbent followed Langmuir isotherm. The adsorption kinetic of dyes was found to conform to pseudo-second order kinetics.

## Introduction

Removal of dye from colored wastewater using adsorbent is interested because specific substances are transferred from liquid onto solid surface. The traditional adsorbents have some disadvantages such as relatively limited pollutant removal capacity and poor separation ability [1-4].

The pollutant removal using magnetic nanoparticle as adsorbents is an emerging field of water and wastewater treatment [5,6]. The magnetic adsorbents could be separated based on their nanostructures because the ease of direction of magnetization would vary depending on the ordering of atoms in the magnetic structure [5,7]. The use of a magnetic field induces the magnetization of the nanoparticle and thus makes the use of a magnetic force possible, but when the magnetic field is cut of, the magnetization immediately decreases to zero. It is important for the release of nanoparticles after adsorption process [6,8].

Several magnetic materials have been used to remove dyes from aqueous solution [9-12]. Nanoparticles have low adsorption capacity of anionic dyes due to repulsion of the negative charge of nanoparticle surface and anionic dyes. Thus, they should be modified. Liu et al. prepared and characterized ammonium-functionalized silica nanoparticle as a new adsorbent to remove methyl orange from aqueous solution [13]. Absalan et al. modified Fe_3_O_4_ magnetic nanoparticles using ionic liquid and used to remove of reactive red-120 and 4-(2-pyridylazo) resorcinol from aqueous samples [14]. A literature review showed that surface modified zinc ferrite nanoparticle by cetyl trimethylammonium bromide (CTAB) was not used to remove dyes. ZFN was synthesized in previous study and used for photo catalytic degradation of dyes [15]. In this paper, zinc ferrite nanoparticle (ZFN) was synthesized and its surface was modified using CTAB. Dyes were removed using ZFN-CTAB and magnetic ferrite nanoparticle (ZFN). Three direct dyes (Direct Red 23 (DR23), Direct Red 31 (DR31) and Direct Green 6 (DG6)) were used as model compounds. The present work aims to study an appropriate and economic procedure for removal of dyes from water by adsorption on ZFN-CTAB as a magnetic adsorbent. The dye adsorption isotherm and kinetic and effect of operational parameters such as adsorbent dosage, initial dye concentration and salt on dye removal was evaluated in details.

## Experimental

### Chemicals

Direct Red 23 (DR23), Direct Red 31 (DR31) and Direct Green 6 (DG6) were obtained from Ciba (Germany) and used without further purification. The characteristics of dyes were shown in Table 1. Other chemicals were of analytical grade and obtained from Merck.

**Table 1:** The characteristics of dyes

**Chemical name**	**Chemical structure**	**Chemical class**	**Color index**	**λ**_**max **_**(nm)**	**M**_**w **_**(g.mol**^**−1**^**)**
C.I.Direct Red 31	C_32_H_21_N_5_Na_2_O_8_S_2_	Diazo class	13390	523	713.6
C.I.Direct Red 23	C_35_H_25_N_7_Na_2_O_10_S_2_	Diazo class	29160	500	813.7
C.I.Direct Green 6	C_34_H_22_N_8_Na_2_O_10_S_2_	Trisazo class	30295	623	812.7

### Synthesis and characterization of ZFN-CTAB

ZFN (≤80 nm) was synthesized in our laboratory. 4.90 g zinc nitrate (297 g/mol) and 13.4 g iron nitrate (404 g/mol) was dissolved in 50 mL distilled water and added to aqueous mixed solution 4.2 g NaOH in 70 mL distilled water and 3 mL ethylene diamine (ED). This solution was heated at 90°C for 1 h to achieve complete chelation. The powder was calcined on alumina crucible at 500°C for 1 h, with a heating rate of 10°C/min [15].

CTAB (0.4 g) was added to solution containing 10 mL acetone, 125 mL distilled water and 1 g ZFN. The mixture was stirred in a mixer for 1 h. The organo-modified ZFN was separated from the mixture by magnetic force and then was washed with distilled water until free of salts.

Fourier transform infrared (FTIR), scanning electron microscopy (SEM) and X-ray diffraction (XRD) were used to characterize ZFN and ZFN-CTAB. FTIR spectrum (Perkin-Elmer Spectrophotometer Spectrum One) in the range 4000–450 cm^−1^ was studied. The morphological structure of the ZFN-CTAB was examined by SEM using LEO 1455VP scanning microscope. Crystallization behavior was identified by XRD model Siemens D-5000 diffractometer with Cu Kα radiation (λ = 1.5406 A°) at room temperature.

### Batch adsorption procedure

The dye adsorption was done by mixing of adsorbent in 250 mL of a dye solution (50 mg/L) for 60 min. The solution samples were taken at certain time intervals (0, 2.5, 5, 7.5, 10, 15, 20, 30, 40, 50 and 60 min.) and adsorbent particles were separated by magnetic force. The change on the absorbance of all solution samples were monitored and determined at certain time intervals during the adsorption process. At the end of the adsorption experiments, the dye concentration was determined.

UV–vis Perkin-Elmer Lambda 25 spectrophotometer was employed for absorbance measurements of samples. The maximum wavelength (λ_max_) used for determination of residual concentration of DG6, DR23 and DR31 in supernatant solution using UV–VIS spectrophotometer were 623, 500 and 523 nm, respectively. The solution pH was adjusted by adding H_2_SO_4_ or NaOH.

The isotherm and kinetics of dye adsorption on ZFN-CTAB was studied by contacting 250 mL of dye solution with initial dye concentration of 50 mg/L at room temperature (25°C) for 60 min at different ZFN-CTAB dosages (0.4-2 g/L).

The effect of operational parameters such as adsorbent dosage (0.4-2 g/L), initial dye concentration (50–200 mg/L), pH (2–11) and salt (0.02 M of NaHCO_3_, Na_2_CO_3_ and Na_2_SO_4_) on dye removal was investigated.

## Results and discussion

### Characterization of ZFN-CTAB

**Figure 1:**
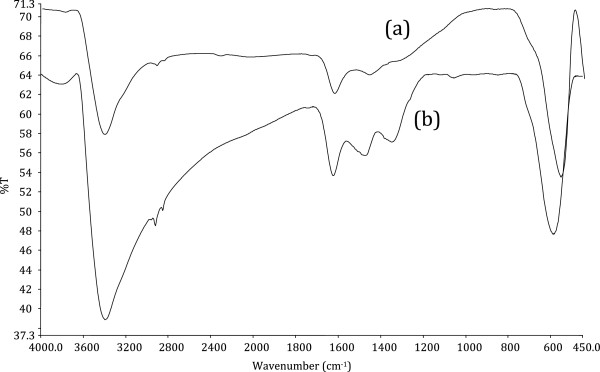
FT-IR spectrum of (a) ZFN and (b) ZFN-CTAB.

**Figure 2:**
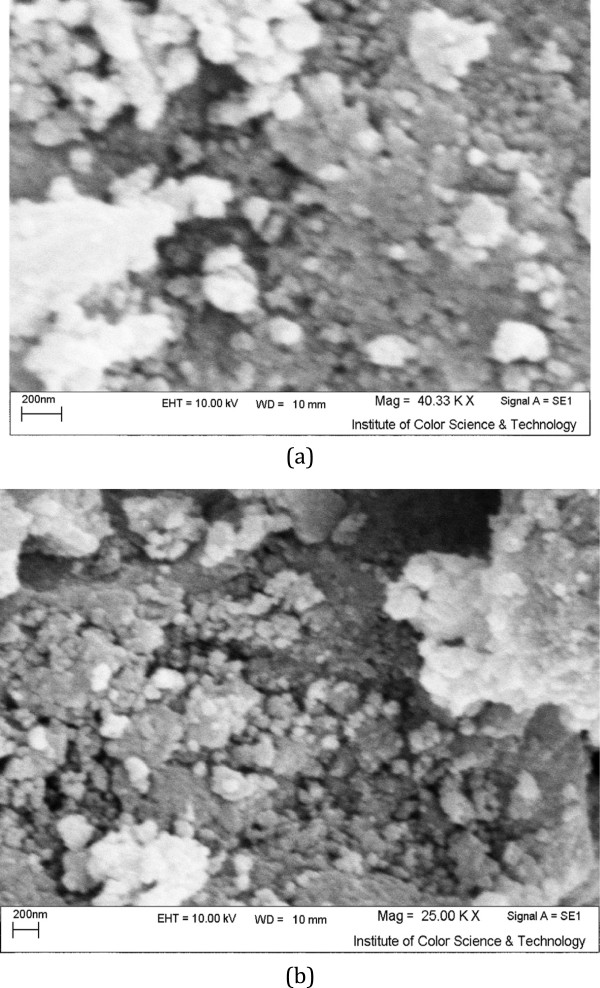
SEM images (a) ZFN and (b) ZFN-CTAB.

The FT-IR spectra of ZFN and ZFN-CTAB were shown in Figure 1. ZFN has two peaks at 3450 cm^−1^ and 500–600 cm^−1^ which indicate O-H stretching vibration and metal-oxygen vibration, respectively. The FTIR spectrum of the ZFN-CTAB displays a number of characteristic bands at 3426 cm^−1^ and 2900–2800 cm^−1^ (Figure 1). These bands are assigned to O-H stretching vibration and -CH_2_- stretching vibration of adsorbed CTAB, respectively [16].SEM is useful for determining the particle shape and appropriate size distribution of the adsorbent. In addition, SEM is a primary tool for characterizing the surface morphology and fundamental physical properties of the adsorbent surface. Scanning electron micrograph of magnetic nanoparticle before and after the modification with the CTAB was shown in Figure 2. The SEM image of samples reveals that ZFN and ZFN-CTAB exhibit a compact arrangement of homogeneous nanoparticles with roughly spherical shape. It can be seen that the particle size of samples are smaller than 80 nm.

**Figure 3:**
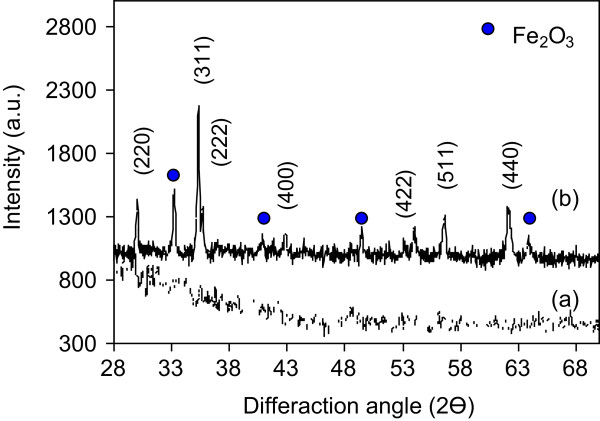
XRD pattern (a) CTAB and (b) ZFN-CTAB.

Figure 3a illustrates the XRD pattern of the CTAB. It has no different characteristic diffraction peaks in the spectrum were observed. The X-ray diffraction pattern of the ZFN-CTAB (Figure 3b) showed that spinel was formed as the most intense (311) peak and Miller indices (220), (222), (400), (422), (511) and (440) matched well with the reflections of the zinc ferrite reported in the previous published paper [17].

### Effect of operational parameter on dye removal

#### Effect of adsorbent dosage

**Figure 4:**
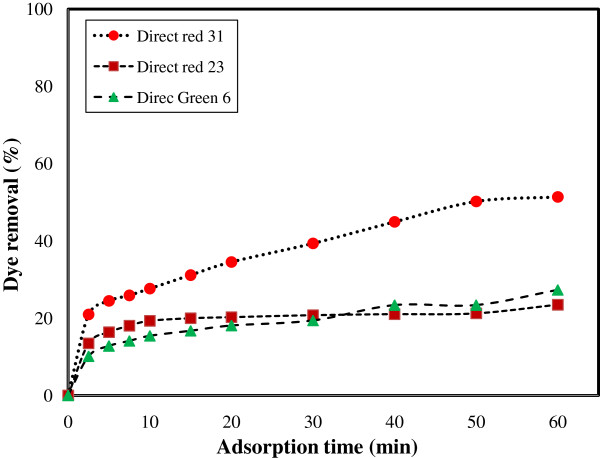
Dye removal by ZFN (without surface modification).

**Figure 5:**
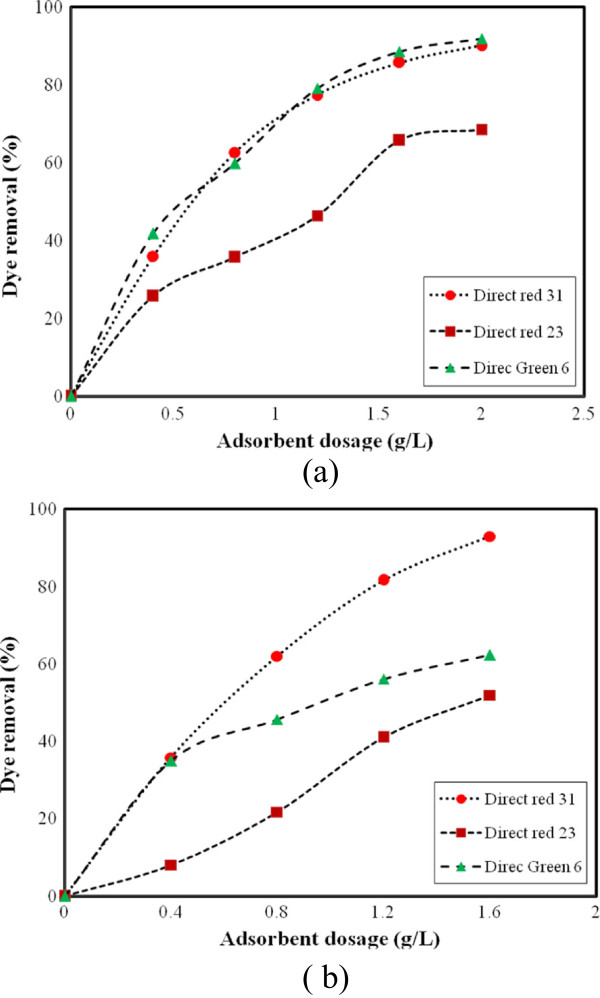
The effect of adsorbent dosage on dye removal by ZFN-CTAB (a) single system and (b) ternary system (Dye concentration: 50 mg/L; pH = 7; adsorption time: 60 min).

The dye removal using ZFN without surface modification (Dye: 50 mg/L, 0.4 g ZFN and pH = 7) was shown in Figure 4. The dye removal efficiency of ZFN was obtained 51, 27 and 23% for DR31, DG6 and DR23, respectively. In additional dye removal using ZFN-CTAB at different adsorbent dosages (g) was shown in Figure 5.

The increase in dye adsorption with adsorbent dosage is due to the increasing of adsorbent surface and availability of more adsorption sites. However, if the adsorption capacity was expressed in mg adsorbed per gram of material, the capacity decreased with the increasing amount of adsorbent. It can be attributed to overlapping or aggregation of adsorption sites resulting in a decrease in total adsorbent surface area available to the dye and an increase in diffusion path length [18]. The results showed that ZFN-CTAB has higher dye removal efficiency in compare with unmodified ZFN. Thus for further study, optimum amount 0.4 g of ZFN-CTAB was used.

#### Effect of dye concentration

**Figure 6:**
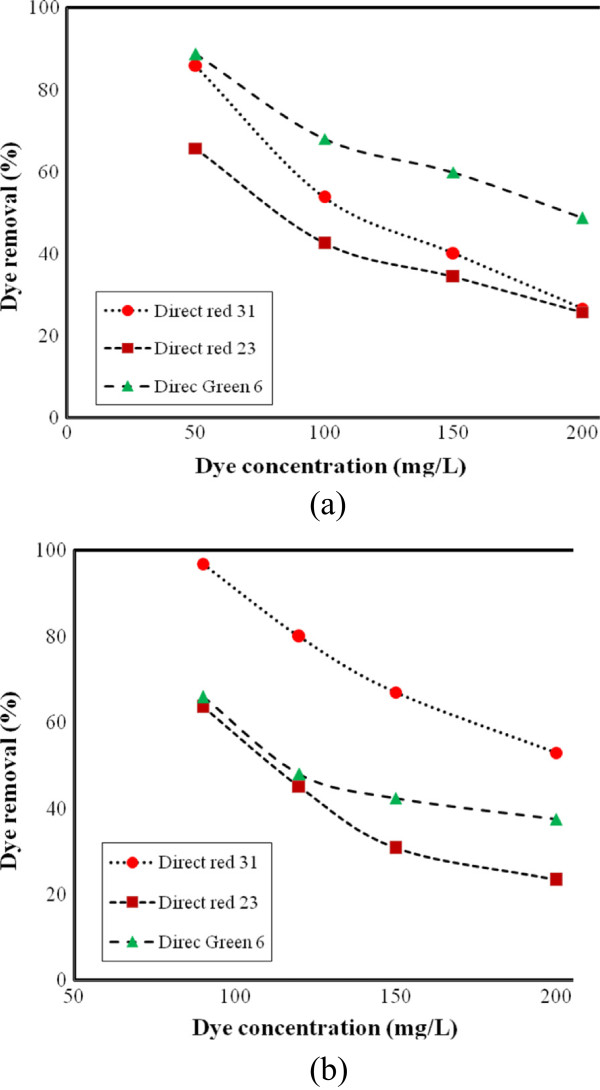
The effect of dye concentration on dye removal by ZFN-CTAB (a) single system and (b) ternary system (adsorbent dosage = 1.6 g/L; pH = 7; adsorption time: 60 min).

Adsorption can generally be defined as the accumulation of material at the interface between two phases [18]. The influence of varying the initial dye concentration of dyes on adsorption efficiencies onto ZFN-CTAB was assessed. The results are shown in Figure 6. It is obvious that the higher the initial dye concentration, the lower the percentage of dye adsorbed. Dye removal of ZFN-CTAB at 50, 100, 150 and 200 mg/L dye concentration was 67, 42 34 and 26% for DR23, 86, 54, 40 and 27% for DR31 and 89, 68, 60 and 49% for DG6, respectively. Furthermore, in ternary system, dye removal of ZFN-CTAB at 90, 120, 150 and 200 mg/L dye concentration was 63, 45, 30 and 23% for DR23, 97, 90, 78 and 45% for DR31 and 51, 48, 42 and 37% for DG6, respectively.

The amount of the dye adsorbed onto ZFN-CTAB increases with an increase in the initial dye concentration of solution if the amount of adsorbent is kept unchanged. It can be attributed to the increase in the driving force of the concentration gradient with the higher initial dye concentration. The adsorption of dye by ZFN-CTAB is very intense and reaches equilibrium very quickly at low initial concentration. At a fixed ZFN-CTAB dosage, the percentage of adsorption decreased. In other words, the residual dye concentration will be higher for higher initial dye concentrations. In the case of lower concentrations, the ratio of initial number of dye moles to the available adsorption sites is low and subsequently the fractional adsorption becomes independent of initial concentration [18].

#### Effect of pH

**Figure 7:**
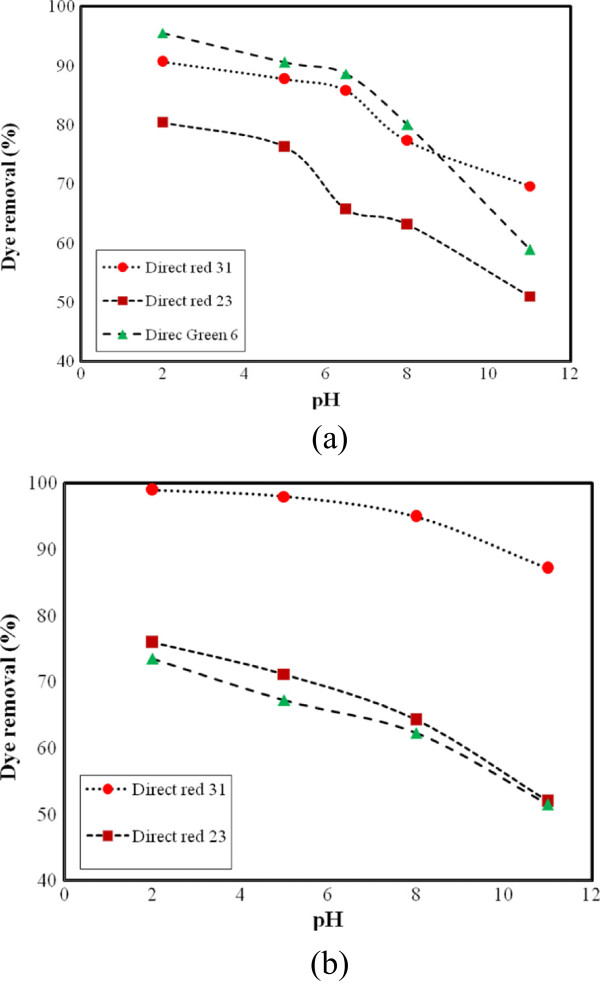
The effect of pH on dye removal by ZFN-CTAB (a) single system and (b) ternary system (Dye concentration: 50 mg/L; adsorbent dosage = 1.6 g/L; adsorption time: 60 min).

The effect of pH on the adsorption of dyes onto ZFN-CTAB is shown in Figure 7. At various pH values, the electrostatic attraction as well as the organic property and structure of dye molecules and ZFN-CTAB could play very important roles in dye adsorption on ZFN-CTAB. At pH 2, a significantly high electrostatic attraction exists between the positively charged (−N+) surface of the ZFN-CTAB and negatively charged anionic dyes. As the pH of the system increases, the number of positively charged sites decreased. It does not favor the adsorption of anionic dyes onto ZFN-CTAB.

#### Effect of salt

The inorganic anions exist in colored industrial wastewater [19]. These substances may compete for the active sites on the adsorbent surface or deactivate the adsorbent. Thus, dye adsorption efficiency decreases.

**Figure 8:**
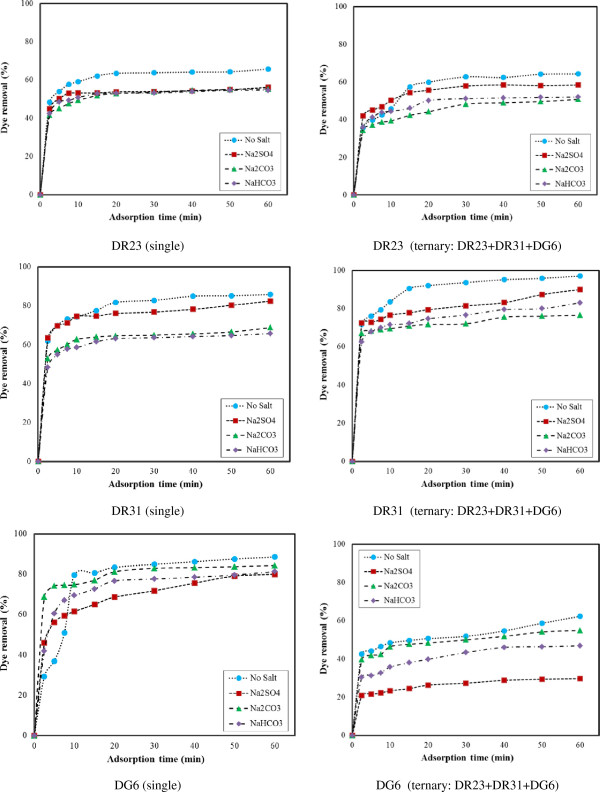
The effect of salt on dye removal by ZFN-CTAB (Dye concentration: 50 mg/L; adsorbent dosage = 1.6 g/L; pH = 7).

To investigate inorganic salts effect on dye removal efficiency, 0.02 M of NaHCO_3_, Na_2_CO_3_ and Na_2_SO_4_ were used. Figure 8 illustrates that dye removal efficiency of ZFN-CTAB is decreased in the presence of inorganic salts because these salts have small molecules and compete with dyes in adsorption by ZFN-CTAB.

### Comparison of single and ternary systems

The results obviously showed that DR31 was removed more than other dyes in ternary system for all effects. The study of dyes adsorption demonstrated that the percentage of adsorption decreased in ternary system (150 ppm) in compare with single system (50 ppm) for each dyes; because some of adsorption sites occupies with other dyes. Investigating of other effect showed that adsorption of dyes had same procedure in both single and ternary systems.

### Adsorption isotherm

The adsorption isotherm is important to design of adsorption systems. The mechanism of dye removal was studied by isotherm models. The relation between the mass of the dye adsorbed at a particular temperature, the pH, particle size and liquid phase of the dye concentration is discussed by the adsorption isotherms. The current research presents a method of direct comparison of the isotherm fit of several models to enable the best-fit and best isotherm parameters to be obtained [20-22]. Several isotherms such as Langmuir, Freundlich and Tempkin models were studied in details [23-25].

The Langmuir isotherm explains the adsorption of dye into adsorbent. A basic assumption of the Langmuir theory is that adsorption takes place at specific sites within the adsorbent [26-29]. The Langmuir equation can be written as follows:

(1) $$q=Q_{0}K_{L}C_{e}/\left(1+K_{L}C_{e}\right)$$

where *q*_*e*_*, C*_*e*_, *K*_*L*_ and *Q*_*0*_ are the amount of dye adsorbed at equilibrium (*mg/g*), the equilibrium concentration of dye in solution (*mg/L*), Langmuir constant (*L/g*) and the maximum adsorption capacity (*mg/g*), respectively.

The linear form of Langmuir equation is:

(2) $$C_{e}q_{e}=1/K_{L}Q_{0}+C_{e}/Q_{0}$$

The Freundlich isotherm was developed mainly to allow for an empirical account of the variation in adsorption heat with concentration of an adsorbate (vapor or solute) on an energetically heterogeneous surface.

Isotherm data were tested with Freundlich isotherm that can be expressed by [26]:

(3) $$q_{e}=K_{F}{C_{e}}^{1/n}$$

where *K*_*F*_ is adsorption capacity at unit concentration and *1/n* is adsorption intensity.

Eq. (4) can be rearranged to a linear form:

(4) $$\log q_{e}=\log K_{F}+\left(1/n\right)\log C_{e}$$

The Tempkin isotherm considered the effects of indirect the heat of adsorption of all the adsorbate molecules on the adsorbent surface layer would decrease linearly with coverage due to adsorbate –adsorbate interactions. The Tempkin isotherm is given as [30]:

(5) $$q_{e}=\mathrm{RT}/\mathrm{bln}\left(K_{T}C_{e}\right)$$

which can be linearized as:

(6) $$q_{e}=B_{1}\ln K_{T}+B_{1}\ln C_{e}$$

Where

(7) $$B_{1}=\mathrm{RT}/b$$

*K*_*T*_ is the equilibrium binding constant (L/mg) corresponding to the maximum binding energy and constant *B*_*1*_ is related to the heat of adsorption.

The parameter values related to Langmuir, Freundlich and Tempkin isotherms were calculated from the slope and intercept of the plots (C_e_/q_e_ vs. C_e_), (log q_e_ vs. log C_e_) and (q_e_ vs. lnC_e_), respectively. The values of *Q*_*0*_*, K*_*L*_*, K*_*F*_*, 1/n, K*_*T*_*, B*_*1*_ and *R*^*2*^ are shown in Table 2.

**Table 2:** **Linearized isotherm coefficients for dye adsorption onto ZFN-CTAB (Q**_**0**_**: mg/g; K**_**L**_**: L/mg; K**_**F**_**: L/g; K**_**T**_**: mg/L and B**_**1**_**: mg.g**^**−1**^**)**

**Dye**	**Langmuir**			**Freundlich**			**Tempkin**		
	**Q**_**0**_	**K**_**L**_	**R**^**2**^	**K**_**F**_	**n**	**R**^**2**^	**K**_**T**_	**B**_**1**_	**R**^**2**^
DR23	26.1100	0.1444	0.9265	10	4.4823	0.4867	4.2018	4.3858	0.4913
DR31	55.5560	0.1314	0.9989	13	2.6896	0.9900	1.2884	12.1790	0.9988
DG6	64.1030	0.1052	0.9013	15	2.8336	0.8991	1.2057	13.4410	0.8411

The *R*^*2*^ values show that the dye adsorption isotherm using ZFN-CTAB does not follow the Freundlich and Tempkin isotherms (Table 2). The linear fit between the *C*_*e*_*/q*_*e*_ versus *C*_*e*_ and the calculated *R*^*2*^ values for Langmuir isotherm model show that the dyes adsorption isotherm can be approximated as Langmuir model (Table 2). This means that the adsorption of dyes takes place at specific homogeneous sites and a one layer adsorption onto ZFN-CTAB surface. The maximum adsorption capacity (Q_0_) was 26.1, 55.5 and 64.1 mg/g for DR23, DR31 and DG6, respectively.

The maximum adsorption capacity of several adsorbents was shown in Table 3. The results show that ZFN-CTAB has high dye adsorption capability. Thus ZFN-CTAB can be used as an adsorbent to remove dyes.

**Table 3:** The dye removal ability of different magnetic adsorbents

***Magnetic adsorbent***	***Dye***	***Q***_***0 ***_ ***(mg/g)***	***Ref.***
Magnetic alginate bead	Methylene blue	9.1	[10]
	Methyl orange	7.9	
Magnetic multi-wall carbon nanotube nanocomposite	Methylene blue	11.9	[11]
	Neutral red	9.8	
	Brilliant cresyl blue	6.3	
γ-Fe_2_O_3_	Acridine orange	59.0	[12]
Multi-walled carbon nanotube filled with Fe_2_O_3_	Methylene blue	42.3	[13]
	Neutral red	77.5	
ZFN-CTAB	Direct Red 23	26.1	This study
	Direct Red 31	55.5	
	Direct Green 6	64.1	

### Adsorption kinetic

The adsorption rate of dyes onto adsorbent was investigated by adsorption kinetics. Adsorption kinetic using pseudo-first order equation, pseudo-second order equation and intraparticle diffusion model were determined in order to investigate the mechanism of dye adsorption onto different adsorbents [31,32].

A linear form of pseudo-first order model (Eq. 8) is [31]:

(8) $$\log\left(q_{e}-q_{t}\right)=\log\left(q_{e}\right)-\left(k_{1}/2.303\right)t$$

where *q*_*e*_*, q*_*t*_ and *k*_*1*_ are the amount of dye adsorbed at equilibrium (*mg/g*), the amount of dye adsorbed at time *t* (mg/g) and the equilibrium rate constant of pseudo-first order kinetics (*1/min*), respectively. The linear fit between the *log (q*_*e*_*–q*_*t*_*)* and contact time (*t*) can be approximated as pseudo-first order kinetics.

Linear form of pseudo-second order model (Eq. 9) was illustrated as:

(9) $$t/q_{t}=1/k_{2}{q_{e}}^{2}+\left(1/q_{e}\right)t$$

where *k*_*2*_ is the equilibrium rate constant of pseudo-second order (*g/mg min*).

The possibility of intraparticle diffusion resistance affecting adsorption was explored by using the intraparticle diffusion model as:

(10) $$q_{t}=k_{p}t^{1/2}+I$$

where *k*_*p*_ and *I* are the intraparticle diffusion rate constant and intercept, respectively.

To understand the applicability of the kinetics models for the dye adsorption onto ZFN-CTAB, linear plots of *log(q*_*e*_*-q*_*t*_*)* versus contact time (*t*), *t/q*_*t*_ versus contact time (*t*) and *q*_*t*_ against *t*^1/2^ are plotted. The values of *k*_*1*_, *k*_*2*_, *k*_*p*_, *I*, *R*^*2*^ (correlation coefficient values) and the calculated *q*_*e*_ (*(q*_*e*_*)*_*Cal*_*.*) are shown in Table 4.

**Table 4:** **Linearized kinetic coefficients for dye adsorption onto ZFN-CTAB (Adsorbent: g; (q**_**e**_**)**_**Exp**_**: mg/g; (q**_**e**_**)**_**Cal.**_**: mg/g; k**_**1**_**: 1/min; k**_**2**_**: g/mg min and k**_**p**_**: mg/g min**^**1/2**^**)**

**Adsorbent**	**(q**_**e**_**) **_**Exp**_	**Pseudo-first order**			**Pseudo-second order**			**Intraparticle diffusion**		
		**(q**_**e**_**)**_**Cal.**_	**k**_**1**_	**R**^**2**^	**(q**_**e**_**)**_**Cal.**_	**k**_**2**_	**R**^**2**^	**k**_**P**_	**I**	**R**^**2**^
DR23										
0.1	32.2091	17.3181	0.0398	0.8789	32.7869	0.0069	0.9930	2.2730	14.7100	0.9699
0.2	22.3055	10.7325	0.0666	0.8420	23.5294	0.0120	0.9961	1.7998	10.3330	0.6732
0.3	19.3612	8.1527	0.0880	0.9428	19.8807	0.0029	0.9999	0.9478	13.0380	0.8027
0.4	20.5434	5.3162	0.0629	0.7408	20.7469	0.0437	0.9998	0.7159	15.6400	0.7659
0.5	17.1485	4.7577	0.0504	0.7261	17.2117	0.0434	0.9993	0.5655	12.9710	0.8655
DR31										
0.1	45.0820	16.8229	0.0613	0.8747	45.8716	0.0111	0.9988	1.8189	31.7220	0.9660
0.2	39.2077	15.0349	0.0401	0.7618	44.4444	0.0080	0.9956	1.6918	25.5390	0.9624
0.3	32.2860	15.3462	0.0728	0.9308	33.3333	0.0121	0.9986	1.5646	20.9890	0.9753
0.4	26.8101	10.0531	0.0848	0.9248	27.3973	0.0242	0.9998	1.0847	19.4360	0.8690
0.5	22.5546	4.9522	0.0573	0.7004	22.6244	0.0487	0.9997	0.5737	18.4420	0.8461
DG 6										
0.1	52.3098	34.4826	0.0896	0.9819	55.2486	0.0052	0.9985	3.6718	27.0230	0.9324
0.2	37.4490	24.2103	0.0385	0.8922	38.4615	0.0043	0.9820	2.2826	14.4790	0.9760
0.3	33.0050	26.8102	0.0461	0.9560	36.7647	0.0026	0.9710	3.3849	6.5411	0.9840
0.4	27.6998	16.2368	0.0845	0.8826	30.3951	0.0065	0.9920	2.7662	9.7794	0.6830

The linearity of the plots (*R*^*2*^) demonstrates that pseudo-first order and intraparticle diffusion kinetic models do not play a significant role in the uptake of the dye by ZFN-CTAB (Table 4). The linear fit between the *t/q*_*t*_ versus contact time (*t*) and the calculated *R*^*2*^ values for pseudo-second order kinetics model show that the dye removal kinetic can be approximated as pseudo-second order kinetics (Table 4). In addition, the experimental *q*_*e*_ (*(q*_*e*_*)*_*Exp*_*.*) values agree with the calculated ones (*(q*_*e*_*)*_*Cal*_*.*), obtained from the linear plots of pseudo-second order kinetics (Table 4).

## Conclusion

In this paper, ZFN-CTAB was synthesized and its dye removal ability was investigated. Direct Red 23 (DR23), Direct Red 31 (DR31) and Direct Green 6 (DG6) were used as model compounds. Adsorption kinetic of dyes was found to conform to pseudo-second order kinetics. It was found that dye adsorption onto ZFN-CTAB followed with Langmuir isotherm. It can be concluded that the ZFN-CTAB being as a magnetic adsorbent with high dye adsorption capacity might be a suitable alternative to remove dyes from colored aqueous solutions.

## Competing interests

The authors declare that they have no competing interests.

## Authors’ contributions

NMM carried out the synthesis of adsorbent and adsorption studies, participated in the sequence alignment and drafted the manuscript. JA carried out the synthesis of adsorbent and adsorption studies. DB participated in the sequence alignment and drafted the manuscript. All authors read and approved the final manuscript.
